# The Downregulation of CRIF1 Exerts Antitumor Effects Partially via TP53-Induced Glycolysis and Apoptosis Regulator Induction in BT549 Breast Cancer Cells

**DOI:** 10.3390/cancers16234081

**Published:** 2024-12-05

**Authors:** Shuyu Piao, Seonhee Kim, Giang-Huong Vu, Minsoo Kim, Eun-Ok Lee, Byeong Hwa Jeon, Cuk-Seong Kim

**Affiliations:** Department of Physiology & Medical Science, College of Medicine, Chungnam National University, Daejeon 301-747, Republic of Korea

**Keywords:** CRIF1, BT549 cells, mitochondrial dysfunction

## Abstract

Background/Objectives: Mitochondrial oxidative phosphorylation (OXPHOS) has been exploited as a therapeutic target in cancer treatments because of its crucial role in tumorigenesis. CR6-interacting factor 1 (CRIF1), a mitochondrial ribosomal subunit protein, is essential for the regulation of mitochondrial OXPHOS capacity. However, the mechanism of CRIF1 in triple-negative breast cancer (TNBC) cells remains unclear. Methods/Results: We showed that the downregulation of CRIF1 reduced cell proliferation in the TNBC cell lines MDA-MB-468, MDA-MB-231, and, especially, BT549. In addition, wound scratch and Transwell assays showed that CRIF1 deficiency inhibited the migration and invasion of BT549 cells. CRIF1 downregulation resulted in the suppression of mitochondrial bioenergetics in BT549 cells, specifically affecting the inhibition of OXPHOS complexes I and II. This was evidenced by a decrease in the mitochondrial oxygen consumption rate and the depolarization of the mitochondrial membrane potential. Damage to mitochondria resulted in a lower adenosine triphosphate level and an elevated production of mitochondrial reactive oxygen species. In addition, CRIF1 deficiency decreased hypoxia-inducible factor 1α accumulation, NADPH synthesis, and TP53-induced glycolysis and apoptosis regulator (TIGAR) expression in BT549 cells. These events contributed to G0/G1-phase cell cycle inhibition and the upregulation of the cell cycle protein markers p53, p21, and p16. Transfection with a TIGAR overexpression plasmid reversed these effects and prevented CRIF1 downregulation-induced proliferation and migration reduction. Conclusions: These results indicate that blocking mitochondrial OXPHOS synthesis via CRIF1 may have a therapeutic antitumor effect in BT549 TNBC cells.

## Simple Summary

CR6 interacting factor 1 (CRIF1) plays an essential role in the regulation of mitochondrial function. We provide important details to demonstrate that CRIF1 deletion-induced mitochondrial damage has an antitumor effect via TIGAR induction in BT549 breast cancer cells. CRIF1 deficiency results in OXPHOS dysfunction, which has been investigated as a potential therapeutic target for the treatment of cancer.

## 1. Introduction

Triple-negative (ER, PR, and HER2/neu) breast cancer (TNBC) renders endocrine therapies ineffective because of the loss of these hormone receptors in breast cancer cells. Chemotherapy is considered the most beneficial treatment for patients with TNBC. However, many patients with TNBC develop chemoresistance and other side effects. Therefore, a new therapeutic treatment is needed to further improve patient outcomes.

According to the Warburg effect, tumor cells preferentially utilize glycolysis instead of mitochondrial oxidative phosphorylation (OXPHOS) for generating energy. However, recent studies have shown that OXPHOS is highly associated with recurrence and death in patients with TNBC [1]. An extensively studied strategy for inhibiting OXPHOS has been investigated as a potential therapeutic target [2,3]. Generally, inhibitors can be classified as direct or indirect depending on whether they target OXPHOS components. Metformin, a drug used to treat type 2 diabetes, directly inhibits OXPHOS complex I. Its antitumor effects in breast cancer have been consistently shown, and it is currently undergoing a phase I trial [4]. Other direct OXPHOS inhibitors (atovaquone, VLX600, and BAY87-2243) also inhibit respiratory chain complexes and have shown anti-proliferative effects in various human cancer cells [5,6,7]. Conversely, indirect inhibitors block OXPHOS function or mitochondrial electron transport chain activity by targeting other regulatory molecules, inhibiting electron transport indirectly. Venetoclax, a Bcl-2 inhibitor, inhibits cell respiration via the indirect inhibition of complex II [8]. Compared with OXPHOS inhibitors, however, the suppression of mitochondrial protein synthesis in breast cancer has received little attention.

As a mitochondrial ribosomal protein, CR6-interacting factor 1 (CRIF1) plays a vital role in OXPHOS subunit synthesis [9]. The silencing of CRIF1 prevents the proper synthesis of mitochondrial DNA-encoded OXPHOS polypeptides and disrupts the assembly and insertion of these subunits into the mitochondrial inner membrane [9]. The targeted disruption of CRIF1 has been used to study mitochondrial OXPHOS dysfunction in a number of research fields [10,11]. Moreover, CRIF1 acts as a negative regulator of the cell cycle by binding to CDK2 or Gadd45 family proteins [12,13]. Previous research showed that CRIF1 deficiency leads to the accumulation of misfolded and damaged OXPHOS proteins accompanied by mitochondrial dysfunction in different cell lines [11,14]. Moreover, the deletion of CRIF1 was shown to promote cell cycle arrest in non-TNBC MCF-7 cells (ER+/PR+/HER2−) [15]. Although recent research revealed that CRIF1-CDK2 interface inhibitors improved sensitivity to taxol treatment in TNBC [16], the antitumor effect of CRIF1 on TNBC cell lines has not been reported. Therefore, we hypothesized that the downregulation of CRIF1 may block OXPHOS subunit biogenesis and the cell cycle, resulting in antitumor effects in BT549 breast cancer cells.

## 2. Materials and Methods

### 2.1. Cell Lines and Transfection

Cells were cultured in a complete growth medium consisting of RPMI (MDA-MB-231 cells) or DMEM (MDA-MB-468 and BT-549 cells) with 10% fetal bovine serum (FBS) and 1% antibiotics. Human recombinant insulin (0.01 mg/mL) was added to the culture medium of MDA-MB-468 and BT-549 cells. Transfection procedure for CRIF1 was performed as described previously [15]. TIGAR overexpression was achieved using a TIGAR expression plasmid (pCMV3-TIGAR) or a negative control vector (pCMV3-negative control) obtained from Sino-biological, Inc. (Beijing, China). BT549 cells were plated at a density of 3 × 10^5^ cells per well in 6-well plates and incubated overnight. The cells were transfected with 0.3 ug of TIGAR plasmid DNA using Effectene transfection reagent (Qiagen (Venlo, The Netherlands)) and then incubated for 48 h at 37 °C w 5% CO_2_ incubator.

### 2.2. Oxygen Consumption Rate (OCR) Analysis

After transfection with siCon or siCRIF1 for 24 h, the experimental protocol was carried out as described previously [15], except for the concentration of oligomycin. An amount of 1.5 µg/mL of oligomycin was used in this study.

### 2.3. Cell Cycle Assay by Flow Cytometry

After transfection with siCRIF for 48 h, the cells were harvested, fixed, and stained with propidium iodide and RNase A. A cell cycle analysis was performed using a flow cytometer, as described previously [15].

### 2.4. Immunoblotting

The following primary antibodies were used: TIGAR, p16, p21, and p53 (Santa Cruz Biotechnology, Santa Cruz, CA, USA); ATP5a1, NDUFA9, SDHA, and UQCRC2 (Invitrogen, Carlsbad, CA, USA); β-actin and COX-4 (Cell Signaling Technology, Beverly, MA, USA); CRIF1 (Abcam, Cambridge, UK); and HIF-1α (Cayman Chemical, Michigan, MI, USA). Immunoblotting was conducted according to a protocol described previously [15].

### 2.5. Cell Proliferation Assay

CCK-8 kit (Dojindo, Tokyo, Japan) was assessed to evaluate cell proliferation. CCK-8 was quantitatively determined according to the manufacturer’s protocol as described previously [15].

### 2.6. Wound Healing Assay

Cells were plated in 6-well plates and incubated for 24 h before transfection with siRNA or plasmid DNA for a further 24 h to reach 90–95% confluence. A scratch was performed with a sterile 200 µL micropipette tip and washed with PBS. Cells were then cultured with serum-free medium and incubated for a further 24 h. Cells were photographed to observe the degree of migration. ImageJ was used to quantify the migration area.

### 2.7. Quantitative Real-Time PCR (qRT-PCR)

Total RNA was extracted using Trizol reagent and amplified using primers for human TIGAR, p16, p21, and GAPDH as previously described [15]. The primer sequence for GAPDH was as follows: GAPDH-sense-5′-TGA ACGGGA AGCTCACTG G-3′ and antisense: 5′-TCCACCACCCT GTTGCTGTA-3′.

### 2.8. Transwell Assay

Cells were transfected with siCRIF1 for 24 h. Cells (1 × 10^5^) in 200 μL serum-free medium were seeded in the upper Transwell insert chamber (24-well plates; Corning Inc., Corning, NY, USA), and 10% FBS medium filled the lower chamber. After incubating for a further 24 h, cells adhering to the upper side were removed, and the cells on the lower surface were stained with DAPI for 15 min, and the blue-fluorescent-stained cells were counted under a fluorescence microscope.

### 2.9. ATP Assay

ATP Assay kit (Cat.#ab83355, Abcam, Cambridge, MA, USA) was utilized to quantify ATP levels following the manufacturer’s protocol.

### 2.10. Mitochondrial Membrane Potential Assay

Mitochondrial membrane potential was assessed using tetramethyl rhodamine ethyl ester (TMRE) dye (Invitrogen). An amount of 100 nM of TMRE in medium was added to cells and incubated at 37 °C for 15 min in the dark. The fluorescence intensity of TMRE was measured with a fluorescence reader (Fluoroskan Ascent, Thermo Fisher Scientific) at 530 nm (excitation) and 590 nm (emission).

### 2.11. Mitochondrial ROS Measurement

MitoSOX red fluorescence (Invitrogen, Waltham, MA, USA) was used to measure mitochondrial ROS. After transfection, cells were stained with MitoSOX (3 µM) in HBSS solution for 15 min at 37 °C and then washed twice with PBS. ROS levels were detected using a fluorescence reader (Fluoroskan Ascent, Thermo Fisher Scientific).

### 2.12. NADP/NADPH Measurements

After transfecting cells with siCRIF1 for 48 h, NADP/NADPH levels were measured using a colorimetric kit (BioVision, Milpitas, CA, USA) following the manufacturer’s protocol. BT549 cells were washed with PBS and lysed with NADP/NADPH extraction buffer. The supernatant was collected, and NADP and NADPH levels were monitored using an absorbance reader at 450 nm and calculated from a standard curve.

### 2.13. Statistical Analysis

Data were analyzed using GraphPad Prism 8.0 software (GraphPad Software, San Diego, CA, USA). One-way analysis of variance (ANOVA), followed by Tukey’s post hoc test and Student’s two-tailed unpaired *t* test were used for statistical analysis. Data are presented as the mean ± standard error of the mean (SEM). Differences were considered statistically significant at *p* < 0.05.

## 3. Results

### 3.1. Downregulation of CRIF1 Showed Antitumor Effects in BT549 Cells

**Figure 1:**
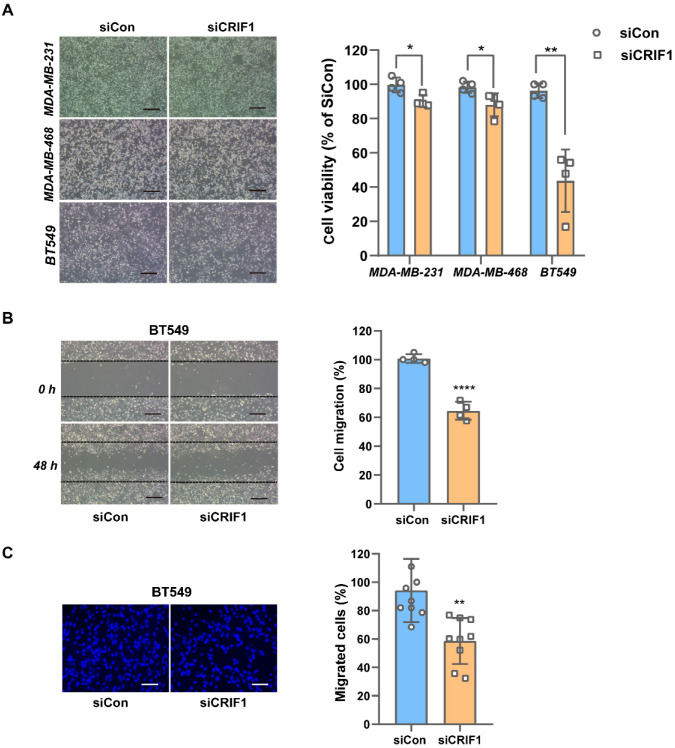
The knockdown of CRIF1 has antitumor effects in BT549 cells. (**A**) Morphological changes in MDA-MB-231, MDA-MB-468, and BT549 cells 48 h after transfection with either siCon or siCRIF1. Cell proliferation was examined using a CCK-8 assay (Bar, 100 µm). (**B**) A wound healing assay was conducted to evaluate the migration of BT549 cells. Images were captured at 0 and 48 h. Quantification of the wound area was performed using ImageJ software (version 1.46) (Bar, 100 µm). (**C**) Cell invasion was evaluated by a Transwell assay (Bar, 50 µm). All data are presented as the mean ± SEM. * *p* < 0.05, ** *p* < 0.01, and **** *p* < 0.0001 versus the siCon group. Con, control; CRIF1, CR6-interacting factor 1; si, small interfering RNA.

To investigate the antitumor effects of CRIF1 deficiency in three TNBC cell lines (MDA-MB-468, MDA-MB-231, and BT549), we transfected the cells with siCRIF1 for 48 h and then examined cell proliferation using the CCK-8 assay. After treatment with 5.5 mM of glucose, all three cell lines showed a significant decrease in proliferation (Figure 1A). However, CRIF1 downregulation had a stronger inhibitory effect on the proliferation of BT549 cells (96.4 ± 4.2% vs. 43.7 ± 18.3%; *p* = 0.001) than on that of MDA-MB-231 cells (99.7 ± 4.3% vs. 90.2 ± 3.4%; *p* = 0.014) and MDA-MB-468 (98.4 ± 3.3% vs. 88.1 ± 6.7%; *p* = 0.033). Therefore, we selected BT549 as the most sensitive cell line and used it for further experiments. We evaluated cell migration ability and observed that CRIF1 deficiency partially inhibited the migration of BT549 cells by wound healing (101.5 ± 4.9% vs. 66.7 ± 7.3%; *p* = 0.000045) and Transwell assays (86.4 ± 13.7% vs. 62.8 ± 15.3%, *p* = 0.0051), respectively (Figure 1B,C). These results indicate that CRIF1 downregulation has an antitumor effect on BT549 cells via the inhibition of cell proliferation and migration.

### 3.2. Downregulation of CRIF1 Inhibited Mitochondrial Function in BT549 Cells

**Figure 2:**
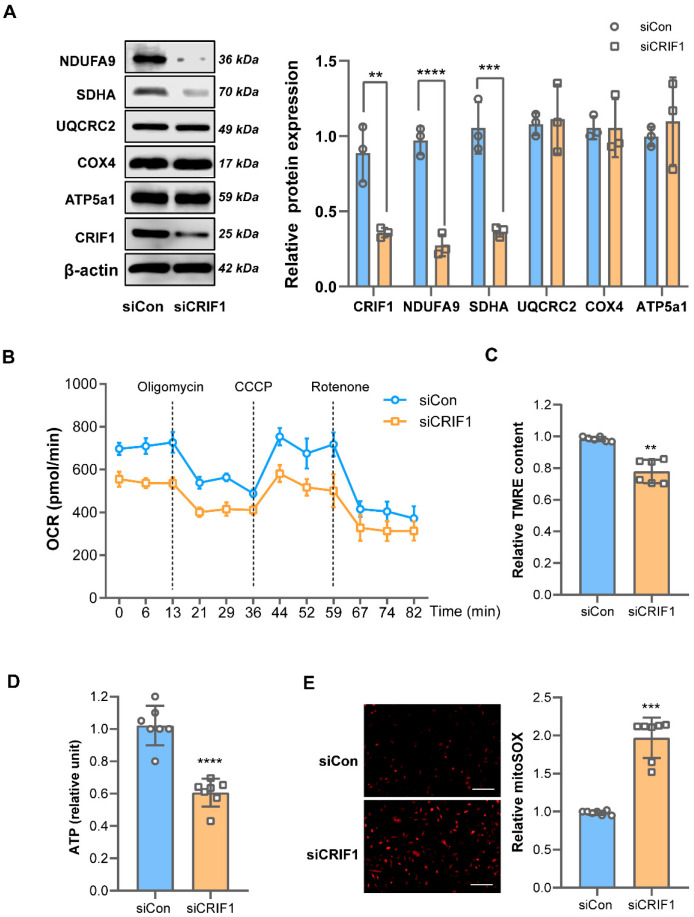
The knockdown of CRIF1 inhibits mitochondrial function in BT549 cells. (**A**) Mitochondrial oxidative phosphorylation complex subunit protein expression was evaluated using a Western blot analysis. (**B**) The OCR was measured with a Seahorse XF analyzer. (**C**) The relative TMRE fluorescence intensity and (**D**) relative ATP levels in siCon and siCRIF1 cells were detected. (**E**) A representative image of MitoSOX red staining (Bar, 100 µm). The MitoSOX red fluorescence intensity was quantified using ImageJ software. All data are presented as the mean ± SEM. ** *p* < 0.01, *** *p* < 0.005, and **** *p* < 0.0001 compared with the siCon group. ATP5a1, ATP synthase F1 subunit α; CCCP, carbonyl cyanide m-chlorophenyl hydrazone; Con, control; COX4, cytochrome c oxidase subunit 4; CRIF1, CR6-interacting factor 1; OCR, oxygen consumption rate; NDUFA9, NADH:ubiquinone oxidoreductase subunit A9; SDHA, succinate dehydrogenase complex flavoprotein subunit A; si, small interfering RNA; TMRE, tetramethyl rhodamine ethyl ester dye; UQCRC2, ubiquinol-cytochrome c reductase core protein 2. Original western blots are presented in File S1.

We previously showed that the downregulation of CRIF1 reduced OXPHOS protein synthesis in several cell lines [11,15], suggesting a possible inhibitory effect of OXPHOS in BT549 cells. Therefore, we evaluated the effect of CRIF1 on mitochondrial function in BT549 cells after the downregulation of siCRIF1 transfection. First, we assessed the protein expression of the OXPHOS complex and the oxygen consumption rate. As shown in Figure 2A,B, CRIF1 downregulation significantly decreased the expression of complex I (NDUFA9) and complex II (UQCRC2) subunits associated with a low mitochondrial oxygen consumption rate via electron transport chain inhibition. These mitochondrial disorders were accompanied by a decreased membrane potential (0.98 ± 0.01% vs. 0.74 ± 0.06%; *p* = 0.002) (Figure 2C), with lower adenosine triphosphate (ATP) levels in CRIF1-deficient cells (1.07 ± 0.08% vs. 0.61 ± 0.10; *p* = 0.00005) (Figure 2D). Given that the downregulation of CRIF1 impaired mitochondrial respiration and ATP generation, disorders of mitochondrial energy metabolism may induce mitochondrial reactive oxygen species (ROS) production. Next, we stained BT549 cells with the mitochondrial ROS dye MitoSOX and observed increased mitochondrial ROS levels in CRIF1-deficient cells (0.98 ± 0.03% vs. 1.91 ± 0.30%; *p* = 0.0006) (Figure 2E). All of these results suggest that CRIF1 deficiency had an inhibitory effect on mitochondrial function in BT549 cells.

### 3.3. Downregulation of CRIF1 Reduced TIGAR Expression and Regulated Cell Cycle Progression

**Figure 3:**
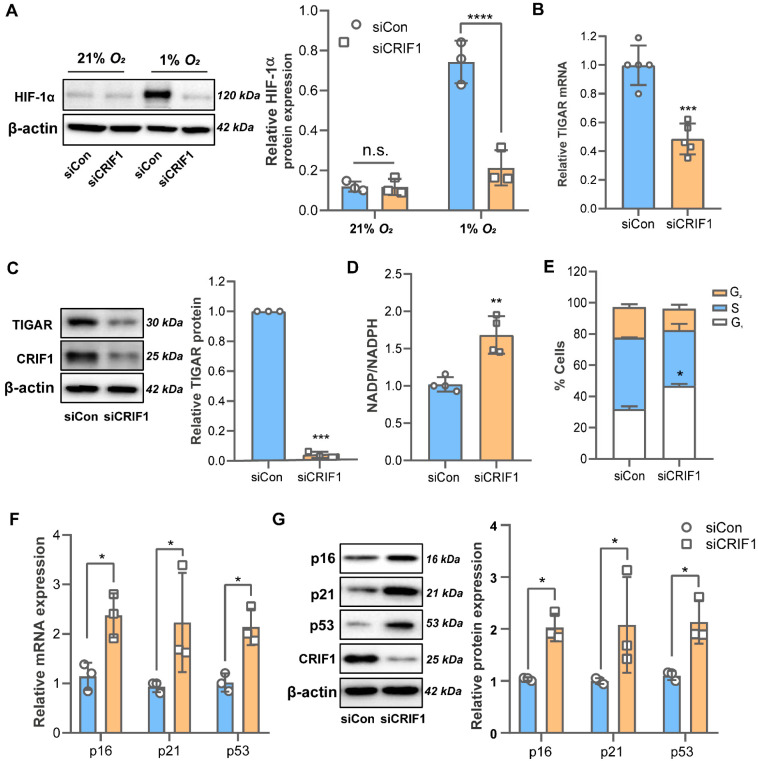
The knockdown of CRIF1 reduces TIGAR expression and regulates cell cycle progression. (**A**) BT549 cells were incubated in a 21 or 1% O2 incubator for 48 h. (**B**) The mRNA level of TIGAR was measured by qRT-PCR. (**C**) TIGAR protein expression was detected by immunoblotting. (**D**) The total NADP/NADPH ratio was measured using a colorimetric/fluorometric assay kit. (**E**) Cell cycle progression was analyzed using flow cytometry. (**F**) mRNA and (**G**) protein levels of p16, p21, and p53 were determined by qRT-PCR and immunoblotting, respectively. All data are presented as the mean ± SEM. * *p* < 0.05, ** *p* < 0.01, *** *p* < 0.005, and **** *p* < 0.0001 compared to the siCon group. n.s., not significant. Original western blots are presented in File S1.

Tumor hypoxia arises as a result of an insufficient oxygen supply and the presence of environmental stress. Hypoxia-inducible factor 1α (HIF-1α), an oxygen-dependent transcription factor, is critical for the adaptive response to hypoxic environments and contributes to cancer cell survival and progression. Mitochondrial dysfunction has been shown to decrease HIF-1α stabilization and improve the effectiveness of hypoxic tumor therapy [17]. Because CRIF1 deficiency impaired mitochondrial function, we evaluated the expression of HIF-1α under normoxic (21% O2) and hypoxic (1% O2) conditions separately. As shown in Figure 3A, under normoxic conditions, there was no difference in HIF-1α levels between the siCon and siCRIF1 groups. Under hypoxic conditions, however, an inhibitory effect was observed in the siCRIF1 group, suggesting that the downregulation of CRIF1 blocked HIF-1α protein accumulation in BT549 cells.

Furthermore, TIGAR is highly expressed in breast cancers and is localized to mitochondria, where it reduces NADPH production. As shown in Figure 3B,C, the downregulation of CRIF1 decreased the mRNA and protein expression of TIGAR, which is crucial for maintaining the mitochondrial NADPH level. Consistent with the reduced TIGAR expression, the NADPH level also decreased via the elevation of the NADP/NADPH ratio (0.96 ± 0.05% vs. 1.65 ± 0.27%; *p* = 0.0027) (Figure 3D). Because TIGAR is proposed to be related to p53-mediated cell cycle regulation [18], we quantified the cell cycle progression status and measured the mRNA and protein levels of three essential cell cycle proteins: p16, p21, and p53. As expected, CRIF1 deficiency induced cell cycle inhibition in the G0/G1 phase (Figure 3E) with increased expressions of p16, p21, and p53 (Figure 3F,G). Taken together, our results indicate that CRIF1 downregulation inhibits TIGAR expression and regulates cell cycle progression.

### 3.4. TIGAR Overexpression Rescued the CRIF1 Deficiency-Induced Antitumor Effect in BT549 Cells

**Figure 4:**
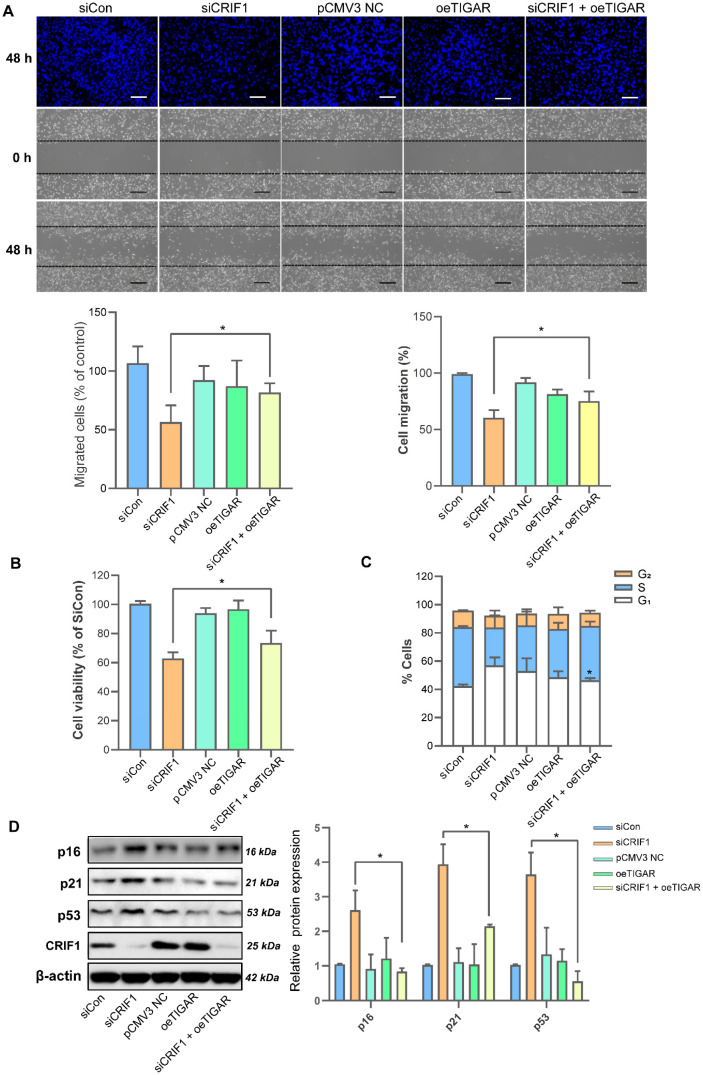
TIGAR overexpression reverses the CRIF1 knockdown-induced antitumor effect in BT549 cells. BT549 cells were transfected with siCon (100 pmol), siCRIF1 (100 pmol), pCMV3 NC (0.3 µg), TIGAR overexpression plasmid (oeTIGAR, 0.3 µg), and siCRIF1 (100 pmol) with TIGAR plasmid (0.3 µg): siCRIF1 + oeTIGAR for 48 h. (**A**) Cell invasion and migration were detected by Transwell and scratch assays, respectively (Bar, 50 µm). (**B**) Cell viability was detected using a CCK-8 assay. (**C**) Cell cycle status was examined using an FACS analysis. (**D**) The protein expression levels of p16, p21, and p53 were detected by immunoblotting. All data are presented as the mean ± SEM. * *p* < 0.05 compared with the siCRIF1 group. Con, control; CRIF1, CR6-interacting factor 1; NC, negative control; si, small interfering RNA; TIGAR, TP53-induced glycolysis and apoptosis regulator. Original western blots are presented in File S1.

To investigate whether TIGAR overexpression rescues cell proliferation and migration in CRIF1-deficient BT549 cells, we generated a TIGAR overexpression plasmid and co-transfected it with siCRIF1 into BT549 cells. As shown in Figure 4A, the elevated expression of TIGAR enhanced the motility of BT549 cells with inhibited migration due to CRIF1 deficiency, as evidenced by both Transwell assays (65.0 ± 12.3% vs. 80.3 ± 8.5%; *p* = 0.022) and wound scratch (67.9 ± 6.9% vs. 83.6 ± 8.5%; *p* = 0.027). In addition, the cell viability assay indicated that TIGAR overexpression in CRIF1-deficient cells markedly enhanced cell proliferation compared with the siCRIF1 group (65.5 ± 3.4% vs. 73.7 ± 7.1%; *p* = 0.012) (Figure 4B). Next, we examined whether TIGAR overexpression affected cell cycle arrest induced by CRIF1 deficiency. As shown in Figure 4C,D, the overexpression of TIGAR inhibited cell cycle arrest in CRIF1-knockdown BT549 cells by reducing the levels of the G1 phase (57.0 ± 4.0% vs. 46.5 ± 1.1%; *p* = 0.015) and cell cycle arrest markers p16, p21, and p53. Taken together, our results indicate that the overexpression of TIGAR suppresses CRIF1 deficiency-induced cell death by inhibiting cell cycle progression.

## 4. Discussion

Although TNBC comprises a small percentage of all breast cancers, it is associated with high mortality and morbidity rates [19,20]. Therefore, there is an urgent need for novel drug treatment strategies. Many FDA-approved drugs, such as arsenic trioxide, metformin, and atovaquone, show anticancer efficacy via the downregulation of OXPHOS in patients with breast cancer [21,22,23]. Therefore, targeting mitochondrial OXPHOS inhibition has been demonstrated to be a promising approach for TNBC treatment. Our study revealed that CRIF1 deficiency inhibited OXPHOS, resulting in mitochondrial dysfunction [11]. This cascade of events led to a reduction in TIGAR expression and cell cycle arrest, ultimately manifesting as antitumor effects in human BT549 TNBC cells.

The loss of CRIF1, a large ribosomal subunit, results in deficiency of the OXPHOS complex and a disturbance in electron transfer, which contribute to mitochondrial dysfunction in various cell lines [10,14]. In addition, CRIF1 was first reported as a cell cycle regulator with therapeutic potential for cancer treatments. Sang et al. demonstrated that combined treatment with selective CRIF1/CDK2 interface inhibitors and paclitaxel (a chemotherapeutic agent) selectively induced apoptotic death in the TNBC cell lines MDA-MB-468 and MDA-MB-231 [16]. However, the antitumor effect of CRIF1 deficiency in BT549 cells has not been investigated until now. In this study, we found that downregulated CRIF1 inhibited the proliferation of three TNBC cell lines, but the percentage of inhibition was nearly five times higher in BT549 cells than in the other cell lines. Therefore, BT549 cells were selected for further analyses. The downregulation of CRIF1 also suppressed the cell migration ability of BT549 cells. Because the knockdown of CRIF1 exerted antitumor effects in vitro, we sought to determine the underlying mechanism.

As a multifunctional factor, CRIF1 has been investigated as a promising target of cancer treatment because it regulates OXPHOS capacity, cell cycle progression, and cell proliferation. CRIF1 is a coactivator of STAT3, a cytoplasmic transcription factor that plays a crucial role in regulating cell proliferation, and interacts with STAT3 via its C-terminal coiled-coil domain. The inhibition of CRIF1 prevents STAT3 activation, leading to the death of blastocysts and prostate cancer cells [24,25]. However, in the HCT116 colon cancer cell line, CRIF1 exerted inhibitory effects on tumorigenesis by increasing p53 gene expression and arresting cell cycle progression [26]. Conversely, CRIF1 is upregulated in hepatocellular carcinoma, contributing to tumor growth and metastasis via the activation of the ROS/NF-κB signaling pathway [27]. In this study, we also evaluated cell cycle modulators (p53, p21, and p16) and their distributions in various populations of cells. In contrast to Yan et al., we found that the inhibition of CRIF1 induced cell cycle arrest by elevating p53, p21, and p16 expression in BT549 cells (Figure 3E–G). We manipulated CRIF1 gene expression to impair mitochondrial function in BT549 cells. Although the downregulation of CRIF1 can reportedly induce mitochondrial dysfunction in different cell lines, the exact effect in BT549 cells has not been investigated until now. In this study, we revealed that CRIF1 deficiency led to a reduction in mitochondrial complexes I and II levels and inhibited mitochondrial respiration, membrane potential, and ATP levels in BT549 cells, which was consistent with the findings for MCF-7 cells in our previous study [15]. However, the downregulation of CRIF1 in MCF-7 cells showed a more severe disruption of mitochondrial function compared to BT549 cells, as indicated by lower OCR, TMRE fluorescence intensity, and ATP levels. Thus, our study indicates that the downregulation of CRIF1 has an anticancer effect that targets both OXPHOS and the cell cycle in BT549 cells.

TIGAR is a fructose-2,6-bisphosphatase that plays an important role in regulating metabolism, ROS, and cancer development. A high expression of TIGAR is observed in the majority of breast cancers, where it contributes to tumor growth and metabolic changes in the tumor microenvironment [28]. Under hypoxic conditions, TIGAR has been reported to translocate to the mitochondria and nucleus to maintain mitochondrial function by reducing ROS production and promoting cell survival via cell cycle regulation. Because CRIF1 is also reportedly involved in mitochondrial dysfunction and cell cycle regulation, we investigated the expression and function of TIGAR in BT549 cells with CRIF1 downregulation. The results show that CRIF1 deficiency significantly inhibited both TIGAR and NADPH levels in BT549 cells, suggesting that TIGAR may also be implicated in inhibiting tumor cell growth and migration. The present study reveals that TIGAR overexpression partially suppresses cell proliferation, migration, and cell cycle arrest triggered by CRIF1 deficiency in BT549 cells. These data suggest that CRIF1 is required for cell growth and migration, which is mediated by TIGAR through its interventions. This study has some limitations in that we exclusively used the BT549 cell line to investigate the antitumor effect of CRIF1 deficiency despite also observing significant reductions in cell viability in both MDA-MB-231 and MDA-MB-468 cells (Figure 1A). It has been documented that the androgen receptor gene is upregulated in BT-549 cells compared to MDA-MB-468 and MDA-MB-231 cells [29]. Furthermore, as the androgen receptor is known to regulate mitochondrial function, it is possible that the stronger inhibitory effect on cell viability observed upon the downregulation of BT549 was related to this mechanism. Further investigations are required to elucidate the mechanism underlying these observations. Another limitation is that we did not observe the changes in mitochondrial morphology and dynamics (fusion and fission), but these will be further investigated in our forthcoming studies.

## 5. Conclusions

**Figure 5:**
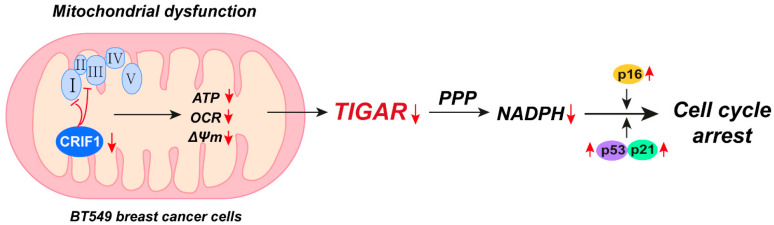
A schematic model summarizing the mechanisms of CRIF1 deficiency in BT549 cells. ΔΨm, mitochondrial membrane potential; CRIF1, CR6-interacting factor 1; OCR, oxygen consumption rate; PPP, pentose phosphate pathway; TIGAR, TP53-induced glycolysis and apoptosis regulator.

These results demonstrate that the inhibition of mitochondrial OXPHOS synthesis by CRIF1 downregulation has antitumor effects via TIGAR induction in BT549 TNBC cells (Figure 5), which has been investigated as a possible approach for cancer therapy.

## Data Availability

The data presented in this study are available upon request from the corresponding author. The data are not publicly available due to ethical and privacy reasons.

## Supplementary Materials

The following supporting information can be downloaded at: https://www.mdpi.com/article/10.3390/cancers16234081/s1, File S1: Original western blots.

## References

[1] Evans K.W., Yuca E., Scott S.S., Zhao M., Paez Arango N., Cruz Pico C.X., Saridogan T., Shariati M., Class C.A., Bristow C.A. (2021). Oxidative Phosphorylation Is a Metabolic Vulnerability in Chemotherapy-Resistant Triple-Negative Breast Cancer. Cancer Res..

[2] Ashton T.M., McKenna W.G., Kunz-Schughart L.A., Higgins G.S. (2018). Oxidative Phosphorylation as an Emerging Target in Cancer Therapy. Clin. Cancer Res..

[3] Molina J.R., Sun Y., Protopopova M., Gera S., Bandi M., Bristow C., McAfoos T., Morlacchi P., Ackroyd J., Agip A.A. (2018). An inhibitor of oxidative phosphorylation exploits cancer vulnerability. Nat. Med..

[4] Saif M.W., Rajagopal S., Caplain J., Grimm E., Serebrennikova O., Das M., Tsichlis P.N., Martell R. (2019). A phase I delayed-start, randomized and pharmacodynamic study of metformin and chemotherapy in patients with solid tumors. Cancer Chemother. Pharmacol..

[5] Zhang X., Fryknas M., Hernlund E., Fayad W., De Milito A., Olofsson M.H., Gogvadze V., Dang L., Pahlman S., Schughart L.A. (2014). Induction of mitochondrial dysfunction as a strategy for targeting tumour cells in metabolically compromised microenvironments. Nat. Commun..

[6] Ellinghaus P., Heisler I., Unterschemmann K., Haerter M., Beck H., Greschat S., Ehrmann A., Summer H., Flamme I., Oehme F. (2013). BAY 87-2243, a highly potent and selective inhibitor of hypoxia-induced gene activation has antitumor activities by inhibition of mitochondrial complex I. Cancer Med..

[7] Fiorillo M., Lamb R., Tanowitz H.B., Mutti L., Krstic-Demonacos M., Cappello A.R., Martinez-Outschoorn U.E., Sotgia F., Lisanti M.P. (2016). Repurposing atovaquone: Targeting mitochondrial complex III and OXPHOS to eradicate cancer stem cells. Oncotarget.

[8] Pollyea D.A., Stevens B.M., Jones C.L., Winters A., Pei S., Minhajuddin M., D’Alessandro A., Culp-Hill R., Riemondy K.A., Gillen A.E. (2018). Venetoclax with azacitidine disrupts energy metabolism and targets leukemia stem cells in patients with acute myeloid leukemia. Nat. Med..

[9] Kim S.J., Kwon M.C., Ryu M.J., Chung H.K., Tadi S., Kim Y.K., Kim J.M., Lee S.H., Park J.H., Kweon G.R. (2012). CRIF1 is essential for the synthesis and insertion of oxidative phosphorylation polypeptides in the mammalian mitochondrial membrane. Cell Metab..

[10] Byun J., Son S.M., Cha M.Y., Shong M., Hwang Y.J., Kim Y., Ryu H., Moon M., Kim K.S., Mook-Jung I. (2015). CR6-interacting factor 1 is a key regulator in Abeta-induced mitochondrial disruption and pathogenesis of Alzheimer’s disease. Cell Death Differ..

[11] Nagar H., Jung S.B., Ryu M.J., Choi S.J., Piao S., Song H.J., Kang S.K., Shin N., Kim D.W., Jin S.A. (2017). CR6-Interacting Factor 1 Deficiency Impairs Vascular Function by Inhibiting the Sirt1-Endothelial Nitric Oxide Synthase Pathway. Antioxid. Redox Signal..

[12] Chung H.K., Yi Y.W., Jung N.C., Kim D., Suh J.M., Kim H., Park K.C., Song J.H., Kim D.W., Hwang E.S. (2003). CR6-interacting factor 1 interacts with Gadd45 family proteins and modulates the cell cycle. J. Biol. Chem..

[13] Ran Q., Hao P., Xiao Y., Xiang L., Ye X., Deng X., Zhao J., Li Z. (2014). CRIF1 interacting with CDK2 regulates bone marrow microenvironment-induced G0/G1 arrest of leukemia cells. PLoS ONE.

[14] Jung S.B., Choi M.J., Ryu D., Yi H.S., Lee S.E., Chang J.Y., Chung H.K., Kim Y.K., Kang S.G., Lee J.H. (2018). Reduced oxidative capacity in macrophages results in systemic insulin resistance. Nat. Commun..

[15] Piao S., Lee I., Kim S., Park H., Nagar H., Choi S.J., Vu G.H., Kim M., Lee E.O., Jeon B.H. (2023). CRIF1 siRNA-Encapsulated PLGA Nanoparticles Suppress Tumor Growth in MCF-7 Human Breast Cancer Cells. Int. J. Mol. Sci..

[16] Sang X., Belmessabih N., Wang R., Stephen P., Lin S.X. (2022). CRIF1-CDK2 Interface Inhibitors Enhance Taxol Inhibition of the Lethal Triple-Negative Breast Cancer. Cancers.

[17] van Gisbergen M.W., Offermans K., Voets A.M., Lieuwes N.G., Biemans R., Hoffmann R.F., Dubois L.J., Lambin P. (2020). Mitochondrial Dysfunction Inhibits Hypoxia-Induced HIF-1alpha Stabilization and Expression of Its Downstream Targets. Front. Oncol..

[18] Madan E., Gogna R., Kuppusamy P., Bhatt M., Pati U., Mahdi A.A. (2012). TIGAR induces p53-mediated cell-cycle arrest by regulation of RB-E2F1 complex. Br. J. Cancer.

[19] Aysola K., Desai A., Welch C., Xu J., Qin Y., Reddy V., Matthews R., Owens C., Okoli J., Beech D.J. (2013). Triple Negative Breast Cancer—An Overview. Hered. Genet..

[20] Howard F.M., Olopade O.I. (2021). Epidemiology of Triple-Negative Breast Cancer: A Review. Cancer J..

[21] Zannella V.E., Dal Pra A., Muaddi H., McKee T.D., Stapleton S., Sykes J., Glicksman R., Chaib S., Zamiara P., Milosevic M. (2013). Reprogramming metabolism with metformin improves tumor oxygenation and radiotherapy response. Clin. Cancer Res..

[22] Dong L.F., Freeman R., Liu J., Zobalova R., Marin-Hernandez A., Stantic M., Rohlena J., Valis K., Rodriguez-Enriquez S., Butcher B. (2009). Suppression of tumor growth in vivo by the mitocan alpha-tocopheryl succinate requires respiratory complex II. Clin. Cancer Res..

[23] Ashton T.M., Fokas E., Kunz-Schughart L.A., Folkes L.K., Anbalagan S., Huether M., Kelly C.J., Pirovano G., Buffa F.M., Hammond E.M. (2016). The anti-malarial atovaquone increases radiosensitivity by alleviating tumour hypoxia. Nat. Commun..

[24] Kwon M.C., Koo B.K., Moon J.S., Kim Y.Y., Park K.C., Kim N.S., Kwon M.Y., Kong M.P., Yoon K.J., Im S.K. (2008). Crif1 is a novel transcriptional coactivator of STAT3. EMBO J..

[25] Tan J.A., Bai S., Grossman G., Titus M.A., Harris Ford O., Pop E.A., Smith G.J., Mohler J.L., Wilson E.M., French F.S. (2014). Mechanism of androgen receptor corepression by CKbetaBP2/CRIF1, a multifunctional transcription factor coregulator expressed in prostate cancer. Mol. Cell Endocrinol..

[26] Yan H.X., Zhang Y.J., Zhang Y., Ren X., Shen Y.F., Cheng M.B., Zhang Y. (2017). CRIF1 enhances p53 activity via the chromatin remodeler SNF5 in the HCT116 colon cancer cell lines. Biochim. Biophys. Acta Gene Regul. Mech..

[27] Chang H., Li J., Qu K., Wan Y., Liu S., Zheng W., Zhang Z., Liu C. (2020). CRIF1 overexpression facilitates tumor growth and metastasis through inducing ROS/NFkappaB pathway in hepatocellular carcinoma. Cell Death Dis..

[28] Ko Y.H., Domingo-Vidal M., Roche M., Lin Z., Whitaker-Menezes D., Seifert E., Capparelli C., Tuluc M., Birbe R.C., Tassone P. (2016). TP53-inducible Glycolysis and Apoptosis Regulator (TIGAR) Metabolically Reprograms Carcinoma and Stromal Cells in Breast Cancer. J. Biol. Chem..

[29] Tseng L.M., Chiu J.H., Liu C.Y., Tsai Y.F., Wang Y.L., Yang C.W., Shyr Y.M. (2017). A comparison of the molecular subtypes of triple-negative breast cancer among non-Asian and Taiwanese women. Breast Cancer Res. Treat..

