# Supplementary figures and images for: The Downregulation of CRIF1 Exerts Antitumor Effects Partially via TP53-Induced Glycolysis and Apoptosis Regulator Induction in BT549 Breast Cancer Cells
Source: Cancers (Basel). 2024 Dec 5;16(23):4081. doi: 10.3390/cancers16234081 (PMC11639960; doi:10.3390/cancers16234081)

Figure 2A

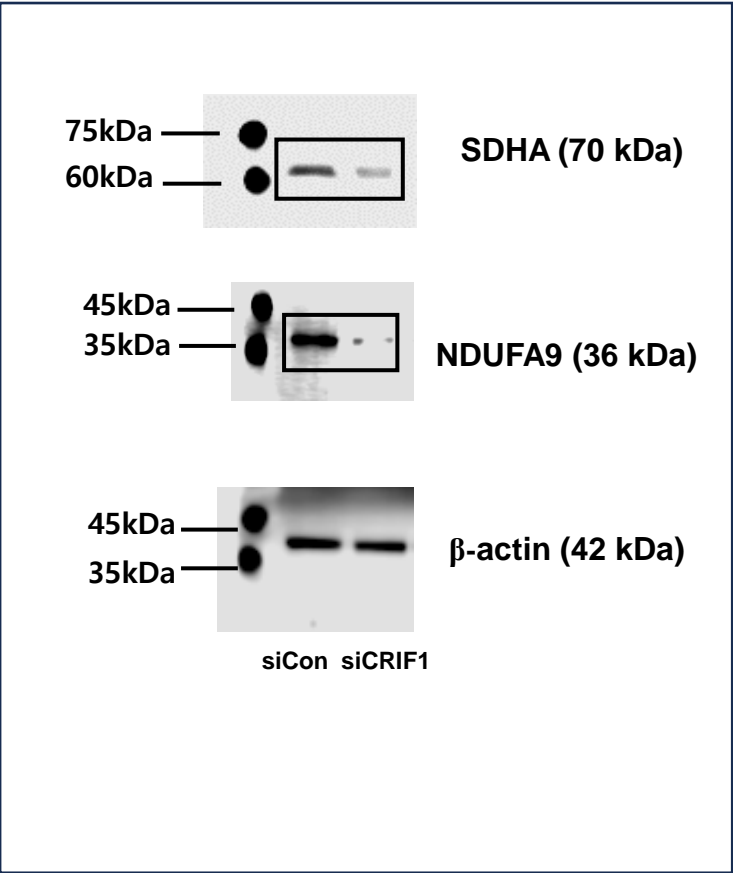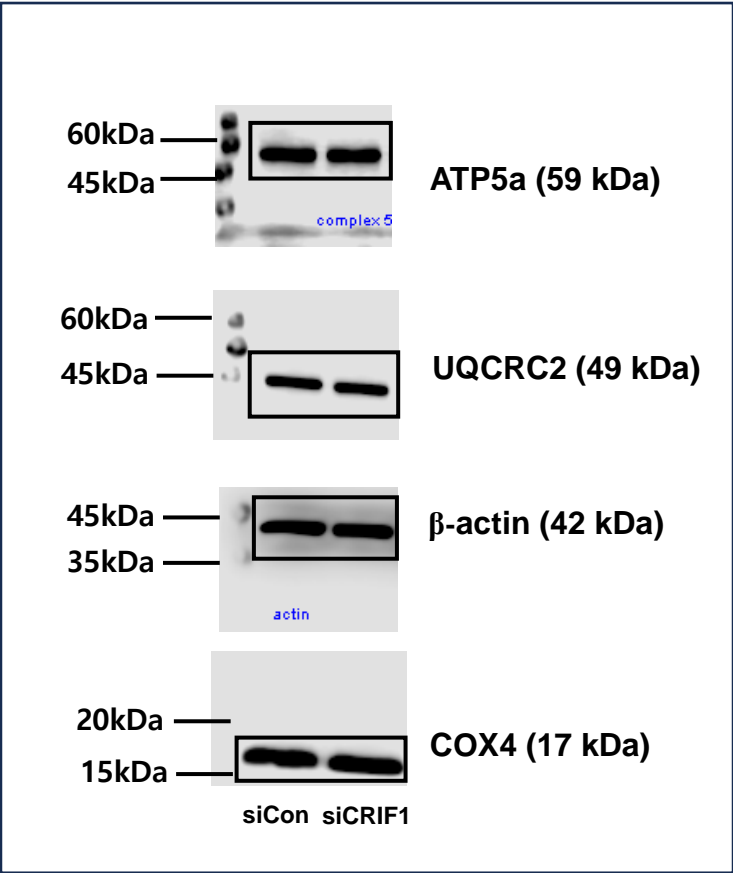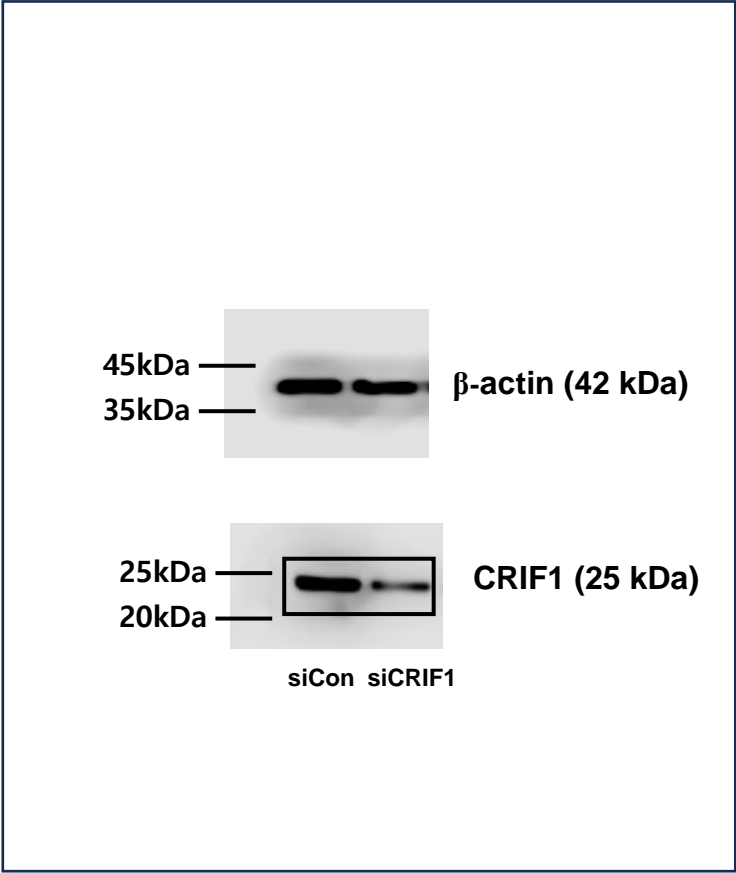

**Figure 3A**

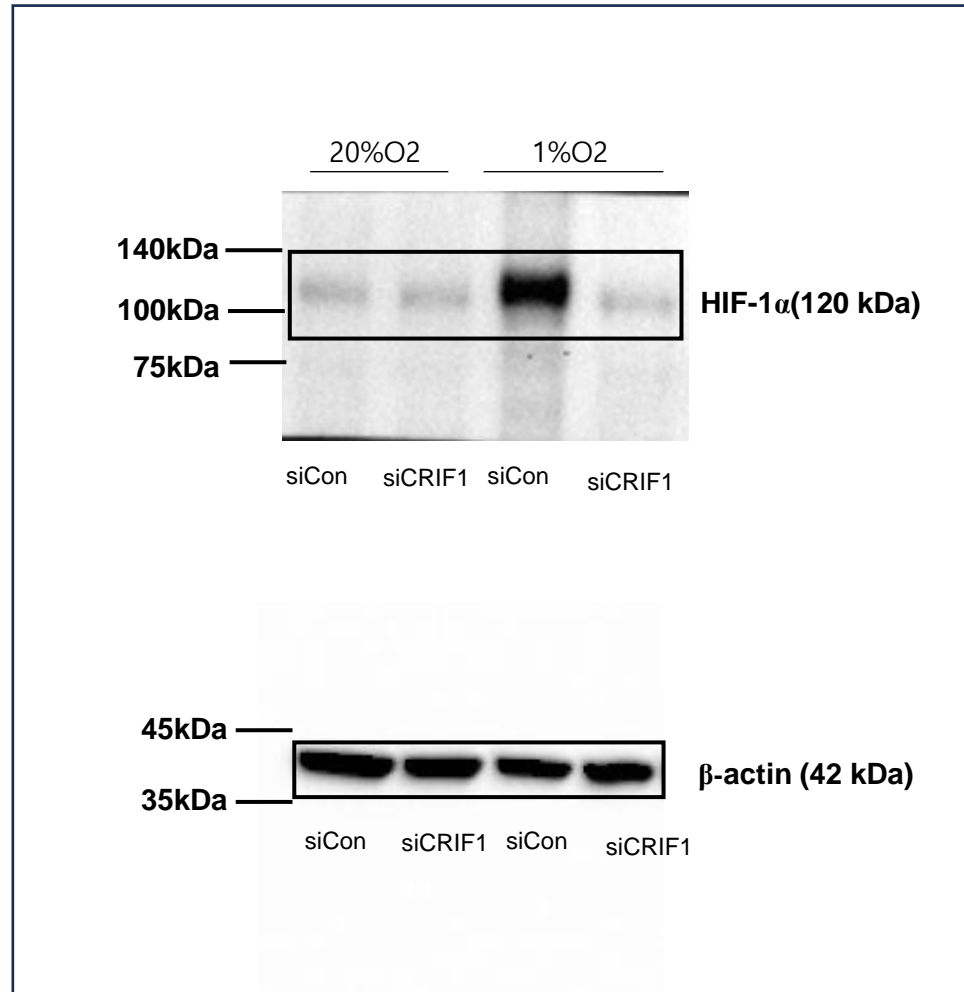

**Figure 3C**

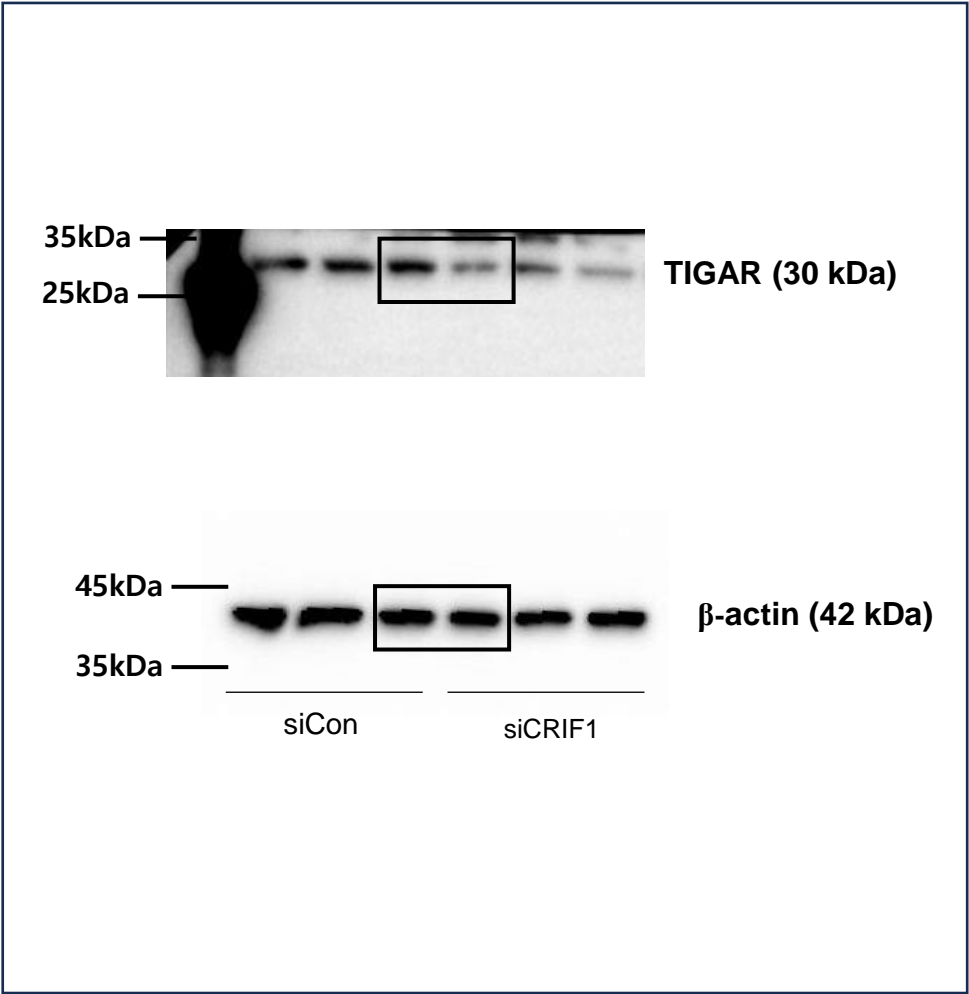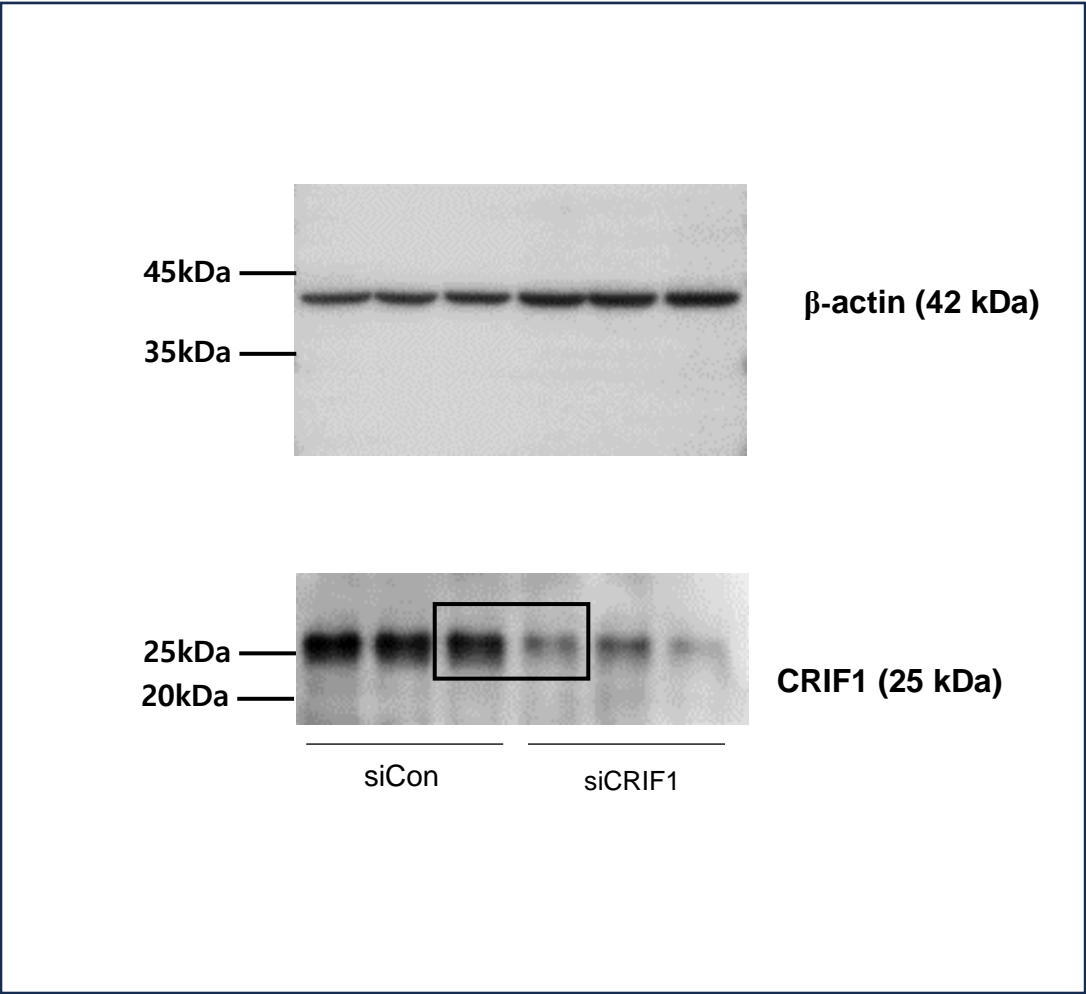

**Figure 3G**

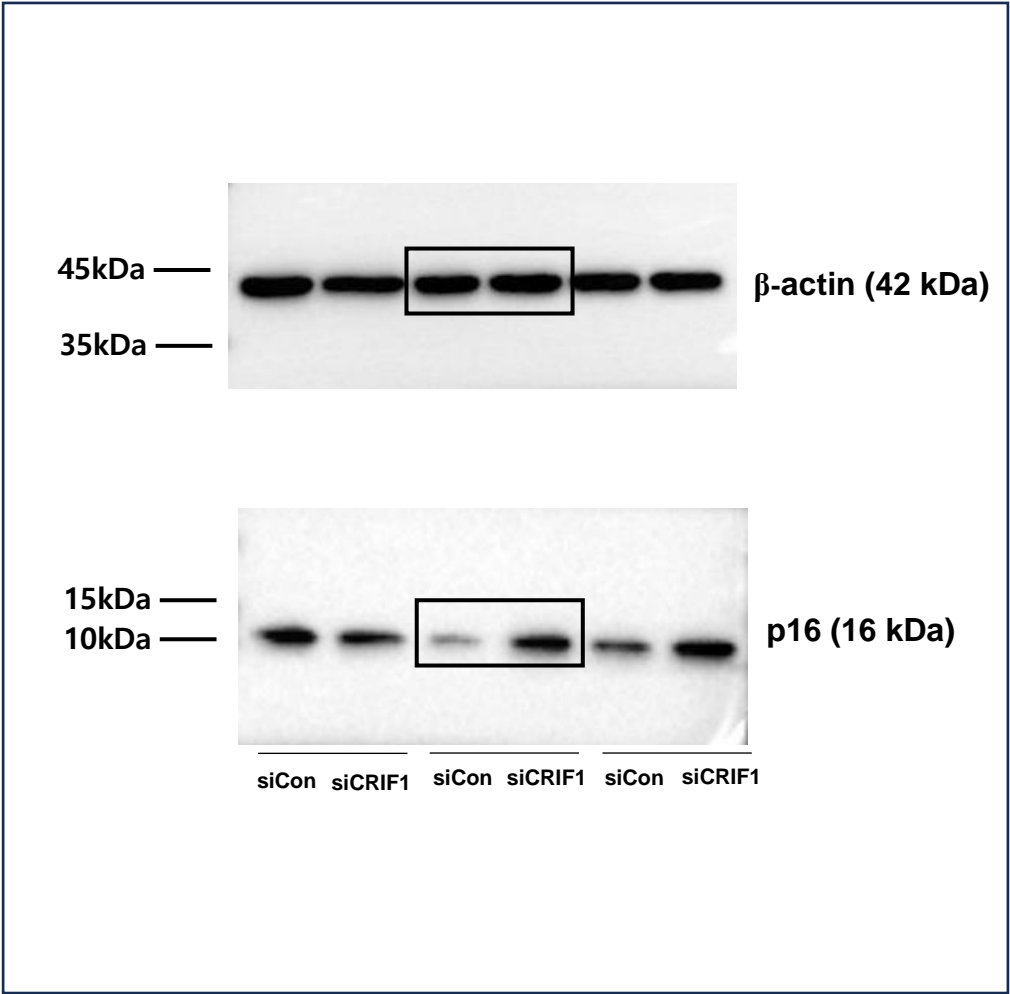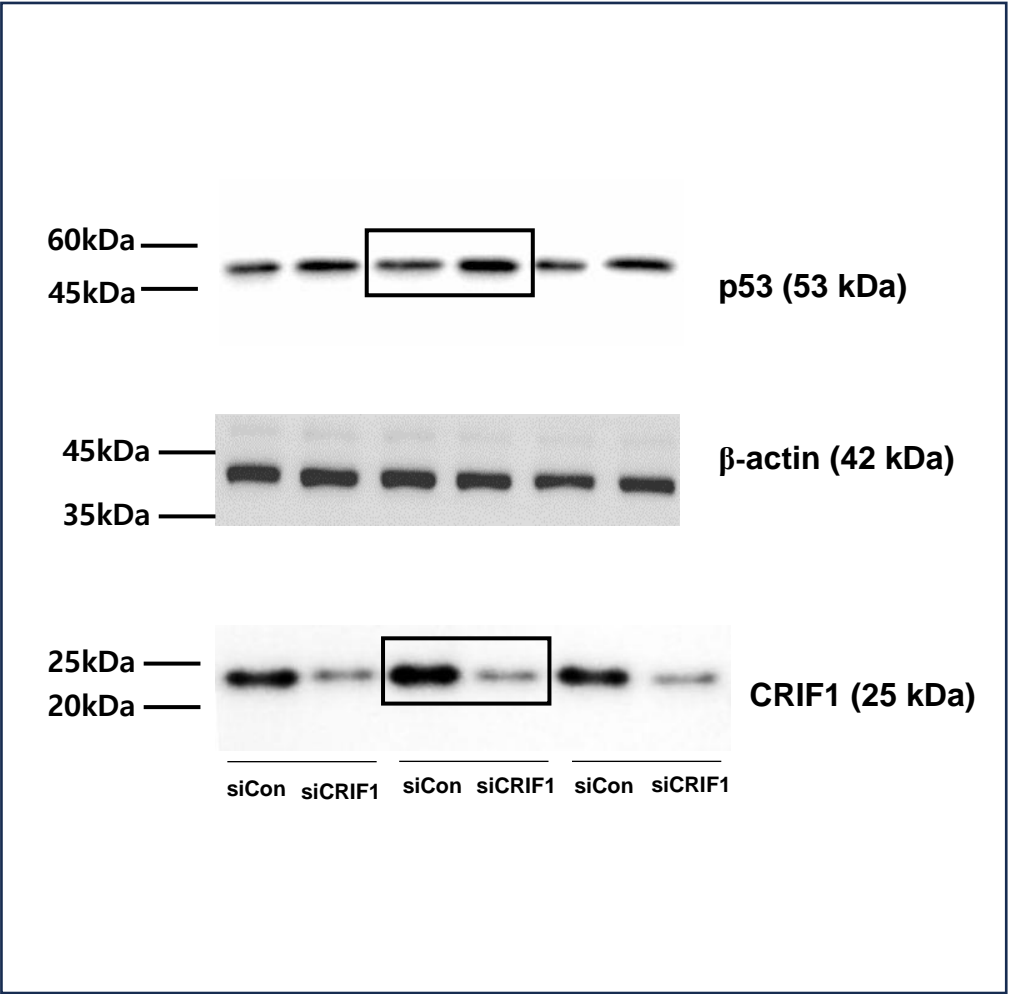

**Figure 3G**

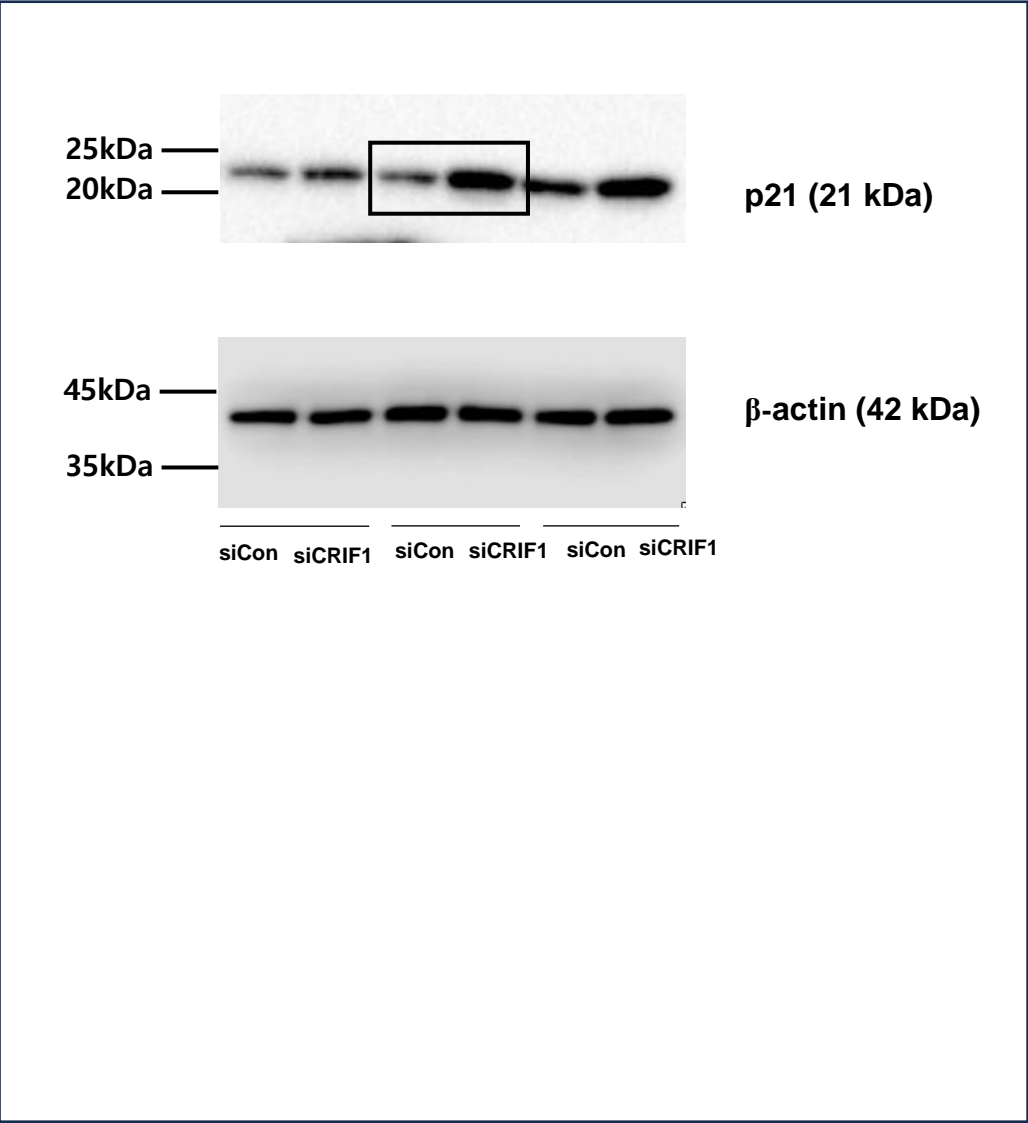

**Figure 4D**

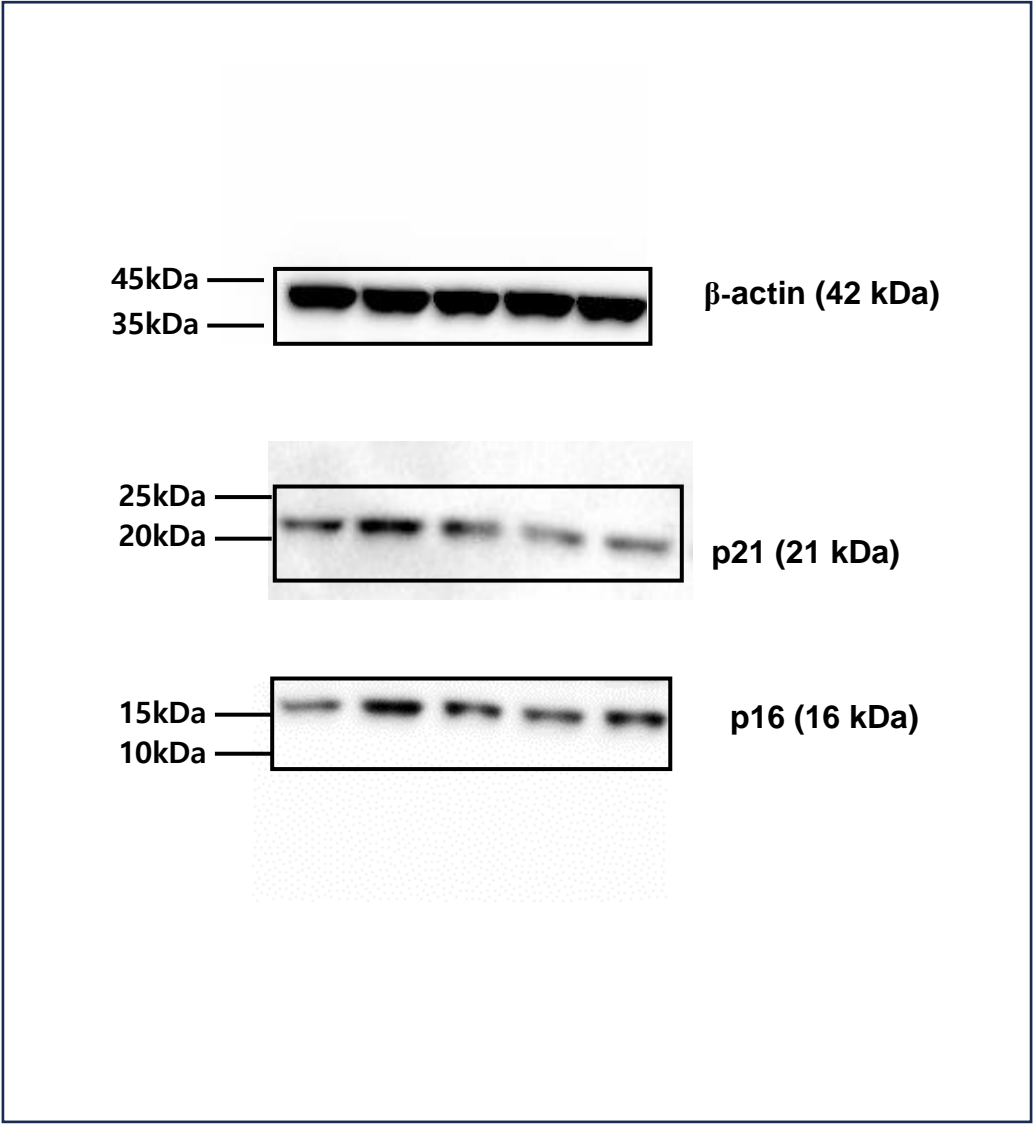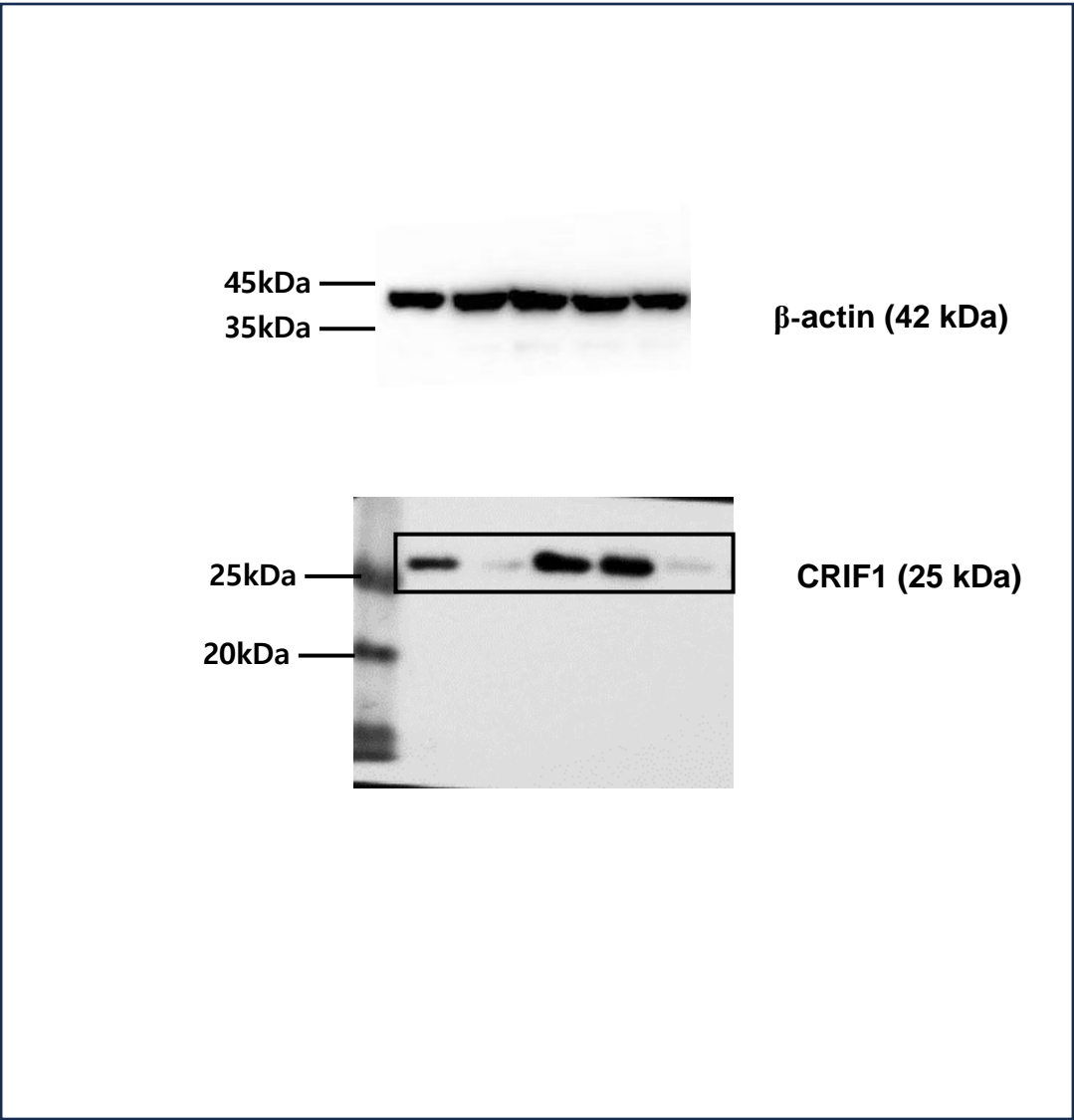

**Figure 4D**

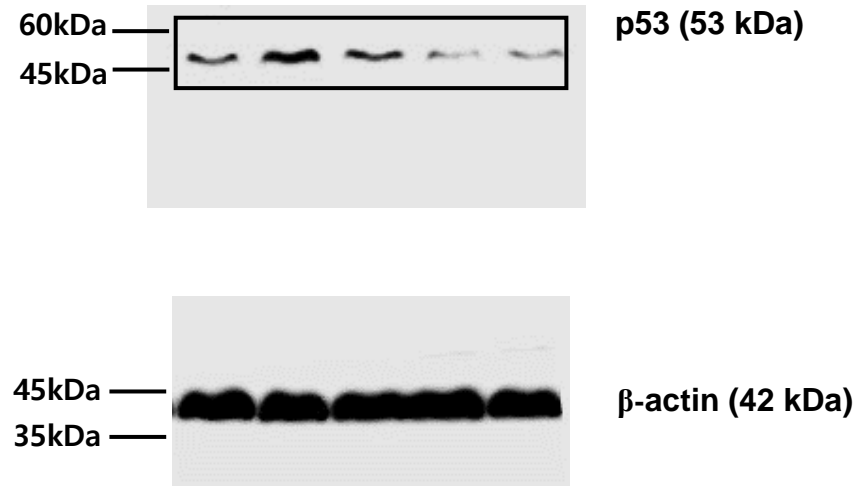

Supplement: Supplementary file 1 [file cancers-16-04081-s001.zip › cancers-3292686-supplementary.pdf]
